# ANCA-Negative Pauci-Immune Glomerulonephritis Associated with Bartonella Endocarditis

**DOI:** 10.1155/2024/4181660

**Published:** 2024-09-06

**Authors:** Camille Ng, Angela Penney, Rojin Sharaflari, Akash Pathak, John H. Howard III, Kuang-Yu Jen

**Affiliations:** ^1^ College of Medicine California Northstate University, Elk Grove, CA, USA; ^2^ Capital Nephrology Medical Group, Sacramento, CA, USA; ^3^ Department of Pathology and Laboratory Medicine University of California Davis Medical Center, Sacramento, CA, USA

## Abstract

Kidney complications can occur due to infective endocarditis, one of which is glomerulonephritis. Most often, an immune complex or complement-mediated glomerulonephritis is seen on kidney biopsy. In a minor subset of cases, pauci-immune glomerulonephritis may be present. Most often, such patients will demonstrate the presence of antineutrophil cytoplasmic antibodies (ANCA) on serologic testing. A growing number of cases of ANCA-associated glomerulonephritis due to Bartonella endocarditis have been reported. This type of endocarditis can present diagnostic difficulties given that these patients are often culture negative. Herein, we report a challenging case of ANCA-negative pauci-immune glomerulonephritis showing florid crescents on biopsy that was associated with Bartonella endocarditis.

## 1. Introduction

Glomerulonephritis is a well-known complication of infective endocarditis, although its frequency has dramatically decreased since the widespread use of antibiotics. Typically, kidney biopsies reveal an immune complex or complement-mediated glomerulonephritis, often with strong C3 staining on immunofluorescence microscopy, which correlates with the frequent finding of low-complement levels on serologic testing [1]. Most often, Staphylococcal or Streptococcal organisms are responsible for the infection [2].

One of the rarer causes of infective endocarditis is Bartonella, which contributes to a significant portion of culture-negative endocarditis [3, 4]. A few case reports have emerged that indicate infective endocarditis due to *Bartonella henselae* can be associated with pauci-immune glomerulonephritis on kidney biopsy [5]. Serologic testing in these patients invariably demonstrates positive antineutrophil cytoplasmic antibodies (ANCA), consisting of antiproteinase 3 (PR3) antibodies. Here, we present an unusual case of ANCA-negative pauci-immune glomerulonephritis secondary to *Bartonella henselae* infective endocarditis and discuss its clinical importance along with its diagnostic challenges.

## 2. Case Presentation

A 57-year-old Laotian man with a remote history of untreated gastric ulcers presented to the emergency department with a 4-month history of epigastric pain, loss of appetite, nausea, and vomiting. The patient reported unintentional weight loss of 60 pounds over the past 3 months and pain on urination. He denied melena, hematemesis, and gross hematuria. The patient had not seen a physician in years but visited his primary care physician for similar symptoms a week before. Physical examination revealed a grade 3 of 6 holosystolic murmur at the left sternal border that radiated to the axilla.

Initial laboratory findings (Table 1) were significant for a BUN of 147 mg/dL and a creatinine of 16.37 mg/dL. Urine studies showed hematuria with >20 RBCs/HPF and spot urine protein to the creatinine ratio of approximately 2 g/g. The urine culture was subsequently negative. Hematologic testing revealed a hemoglobin of 5.7 g/dL, (13.5–17.5 g/dL), platelets of 101 K/*µ*L (150–450 K/*µ*L), and schistocytes were present on peripheral smear. Unfortunately, no lactate dehydrogenase or haptoglobin was assessed to determine if microangiopathic hemolytic anemia was present. The international normalized ratio was 1.3, prothrombin time test was within normal range at 29.5 s (22.7–34.8 s), and total bilirubin ranged from 0.5 to 0.7 mg/dL (0.2–1.2 mg/dL).

Initial imaging studies included a computed tomography (CT) scan of the abdomen and pelvis, which demonstrated an incidental indeterminate right hepatic lobe lesion but was otherwise unremarkable. A retroperitoneal ultrasound showed the right kidney at 11.4 cm in length and the left kidney at 11.3 cm in length, both of which were echogenic and without stones or hydronephrosis. Both kidneys were in their normal position and had normal Doppler flow.

The patient underwent an upper endoscopy on day 2 of hospitalization, which showed severe gastritis without mass lesions. On the same day, a transthoracic echocardiogram demonstrated severe regurgitation with a thickened and calcified posterior mitral leaflet that appeared flail with structural damage to the leaflet, concerning for endocarditis. A follow-up transesophageal echocardiogram showed multiple vegetations on the mitral valve, the largest measured up to 2.4 × 2.2 cm. In addition, magnetic resonance imaging and CT scan of the head revealed multiple embolic strokes and small subarachnoid hemorrhages bilaterally. At this point, hemodialysis was initiated due to worsening severe kidney failure, and given the imaging findings of endocarditis, ceftriaxone 2 g IV after dialysis and vancomycin IV (hemodialysis dosing, with a goal trough of 15–20 mcg/mL) were both initiated on day 3. The patient was not deemed a surgical candidate by both cardiology and neurosurgery due to his poor nutritional status, severe anemia requiring 5 units of packed red blood cells, thrombocytopenia, and acute kidney injury. Subsequently, the gastric biopsies taken during the upper endoscopy performed on day 2 were reported as positive for *Helicobacter pylori*, and the patient was started on triple therapy on day 6 with pantoprazole 40 mg IV BID, metronidazole 500 mg IV q8 h, and doxycycline 100 mg IV q12 h.

Four sets of blood cultures, collected over approximately 36 hours from admission prior to antibiotic therapy, were negative for growth. However, *Bartonella henselae* IgM antibody titers were elevated at 1 : 128, but Brucella and Q fever antibody titers were negative. Thus, rifampin 600 mg PO daily was added on day 8. Additional serologic tests, including antinuclear, anti-double-stranded DNA, and antiglomerular basement membrane antibodies, were negative. The ANCA titer was <1 : 20, and myeloperoxidase and PR3 levels were negative. C3 and C4 levels were low at 57 mg/dL (90–180 mg/dL) and 9 mg/dL (10–40 mg/dL), respectively.

**Figure 1:**
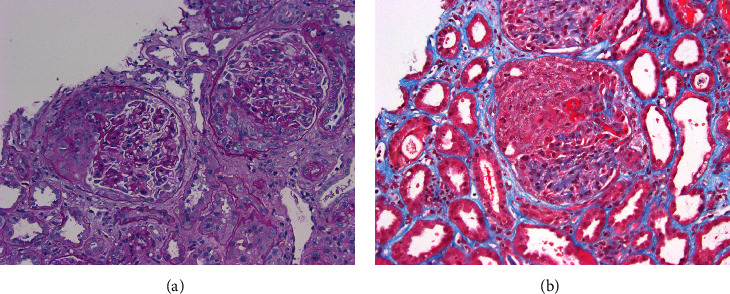
Glomerular findings on kidney biopsy: (a) periodic acid-Schiff stain showing two glomeruli with large cellular crescents (200x magnification); (b) Masson trichrome stain showing a glomerulus with a large cellular crescent in association with fibrinoid necrosis from a necrotizing lesion (200x magnification). Note the background acute tubular epithelial cell injury.

Given the patient's microscopic hematuria, subnephrotic proteinuria, low complement levels, and profound kidney dysfunction, glomerulonephritis was suspected, and a kidney biopsy was performed. The light microscopy portion of the biopsy contained approximately 27 glomeruli, of which one showed global sclerosis. At least 20 of the glomeruli (∼75%) contained cellular crescents, many in association with necrotizing lesions (Figure 1). Unaffected glomeruli appeared relatively unremarkable, showing no significant endocapillary or mesangial hypercellularity. No significant interstitial fibrosis or tubular atrophy were identified. The tubules displayed widespread acute injury. Scattered red blood cell casts were noted as well. Patchy interstitial inflammation composed mostly of mononuclear leukocytes was present. A few scattered foci of neutrophils were observed, focally associated with tubular involvement. The arteries/arterioles showed typical changes of chronic vascular disease, but no frank vasculitis or changes of thrombotic microangiopathy were seen. Aside from internal positive control staining, immunofluorescence microscopy was negative for IgG, IgA, IgM, C1q, C3, kappa light chain, lambda light chain, and albumin. Fibrinogen was noted to demonstrate focal segmental glomerular staining corresponding to necrotizing lesions. Electron microscopy revealed glomerular basement membranes of normal thickness and contours. No electron-dense immune-type deposits, organized deposits, or tubuloreticular inclusions were identified. Podocyte foot processes were mildly to moderately effaced in a patchy distribution. Overall, the biopsy findings were consistent with a diffuse crescentic pauci-immune glomerulonephritis.

Given the presence of an extremely active glomerulonephritis, the patient was started on methylprednisolone 500 mg IV for three days and then switched to 60 mg oral prednisone for 2 weeks followed by a 5-week taper. The patient continued to have anemia and underwent another endoscopy and colonoscopy, which showed diffuse gastritis and benign sigmoid colon polyps, respectively. Due to poor nutritional status and nausea, the metronidazole component of the patient's triple therapy for *H. pylori* was stopped after 5 days of therapy, only continuing the doxycycline for treatment of his Bartonella infection. The patient was discharged from the hospital 18 days after initial admission and was continued on dialysis. He continued to receive ceftriaxone and vancomycin IV dosed after dialysis, doxycycline 100 mg PO twice daily, rifampin 600 mg PO daily, prednisone 50 mg PO daily, and pantoprazole 40 mg PO daily.

Three weeks after discharge, the patient presented back to the emergency department due to acute encephalopathy and dyspnea after his family members found him unresponsive. On arrival, he was febrile, tachycardic, and hypoxic with an oxygen saturation of 80%. An echocardiogram revealed a very large vegetation measuring 3.6 × 2.5 cm on the posterior mitral valve leaflet. Head CT scan revealed a large intracerebral hemorrhage in the right temporal occipital region, likely secondary to septic emboli with hemorrhagic transformation. Two repeated *Bartonella henselae* IgM antibody titers during this second hospital admission were 1 : 32 and 1 : 16, lower than what was seen on his initial presentation. The antibiotic regimen of doxycycline, rifampin, and vancomycin was continued, and ceftriaxone was escalated to meropenem 1 g IV q24 h for culture-negative endocarditis and septic shock, accompanying admission to the intensive care unit.

Over the subsequent 24 hours, the patient stabilized and returned to his baseline cognitive status. After evaluation by cardiothoracic and neurosurgery teams, it was determined again that the patient was not a candidate for operative intervention. Nine days after admission, his respiratory status improved, and he was discharged with no further significant clinical changes and continued on doxycycline 100 mg PO twice daily, rifampin 600 mg PO daily, and prednisone 20 mg PO daily with a taper over 1 week to 10 mg PO daily that he remained on indefinitely.

**Figure 2:**
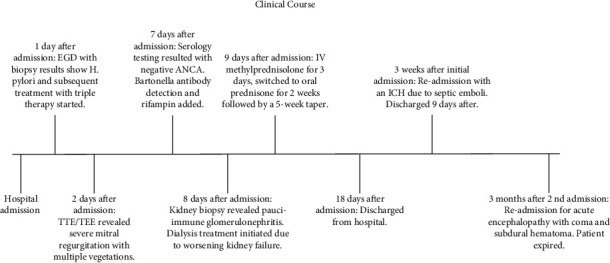
Timeline of clinical course.

The patient was readmitted 3 months later with recurrent acute encephalopathy, which quickly progressed to a coma. Head CT revealed an acute large left subdural hematoma with a midline shift. He rapidly deteriorated and was deemed not to be a surgical candidate by neurosurgery due to loss of pupillary reflexes and anticipated lack of benefit from intervention. He gradually lost brainstem reflexes, became hemodynamically unstable, and ultimately passed away. A timeline of the patient's clinical course is shown in Figure 2.

## 3. Discussion

Infective endocarditis is historically diagnosed through clinical presentation, blood cultures, and echocardiography. The 2023 Duke-International Society for Cardiovascular Infectious Disease (ISCVID) is the newly accepted diagnostic criteria for infective endocarditis, which is an updated version of the 2000 modified Duke criteria [6]. When patients present with vegetations on echocardiogram consistent with endocarditis but lack a clear microbiologic diagnosis, culture negative endocarditis should be suspected. Bartonella endocarditis is one of the most common causes of culture negative endocarditis in the United States [3, 4, 7]. Along with clinical suspicion and echocardiographic evaluation, diagnostic testing includes obtaining at least two blood cultures, serology for *Bartonella* spp., and serum or plasma polymerase chain reaction testing [6–8]. *Bartonella henselae* and *Bartonella quintana* infections are classically associated with cats and human lice, respectively. Although our patient's history did not reveal contact with cats, he did live with dogs at his home, which may have predisposed him to his infection. Unlike our patient, multiple patients who were eventually diagnosed with Bartonella endocarditis had a history of cardiac valve replacement, perhaps indicating a predisposing factor that warrants a further retrospective analysis [9–11].

In our case, Bartonella endocarditis was diagnosed through a combination of clinical presentation and physical exam, echocardiographic findings, and elevated *Bartonella henselae* antibody titers. Our patient had an atypical presentation, as his chief complaints were epigastric pain and gastrointestinal in nature. Fever is the most commonly reported symptom in infective endocarditis [12]; however, our patient remained afebrile throughout his initial hospital stay. Other potential presenting symptoms are often vague, including fatigue, malaise, myalgias/arthralgias, night sweats, chest pain, abdominal pain, and dyspnea [13, 14]. Our patient experienced abdominal pain, anorexia, and weight loss. These were initially attributed to his suspected gastritis but may have also been a result of his underlying endocarditis. Our patient's presentation highlights the importance of the physical examination findings, specifically a cardiac murmur, in supporting a diagnosis of endocarditis. Approximately 75% of patients with infective endocarditis present with a cardiac murmur [12]. Though a murmur is not independently diagnostic of endocarditis, the discovery of a murmur in our case prompted the evaluation through echocardiography, leading to an efficient diagnosis of endocarditis and its related sequelae. Patients who do not maintain long-term healthcare and therefore lack a well-detailed health history would benefit from additional testing to at least set a baseline for cardiac function and perhaps diagnose any underlying pathology that may have gone undetected.

Our patient's clinical course was complicated by severe acute kidney injury that raised the possibility of rapidly progressive glomerulonephritis (RPGN) due to presumed rapid decline in kidney function and the presence of proteinuria and hematuria. RPGN is often associated with autoimmune conditions but can also be secondary to infectious processes such as endocarditis. The clinical suspicion for RPGN requires a kidney biopsy to confirm and specifically classify the glomerulonephritis. Glomerulonephritis in the setting of infective endocarditis, although not common, is well documented and requires a high degree of clinical suspicion and prompt intervention. Multiple previous studies have shown a link between infective endocarditis and glomerulonephritis, with immune complex-mediated glomerulonephritis being most common [1, 15].

In this patient, pauci-immune glomerulonephritis was confirmed on kidney biopsy due to the presence of crescents and necrotizing lesions and the lack of immune deposits on immunofluorescence and electron microscopy. The glomerulonephritis was strikingly active with diffuse crescentic and necrotizing lesions and likely related to the underlying Bartonella infection.  Table 2 details several previously reported cases of Bartonella endocarditis specifically associated with pauci-immune glomerulonephritis, all of which showed ANCA/PR3 positivity. Our report presents a rare case of Bartonella endocarditis associated with pauci-immune glomerulonephritis lacking ANCA positivity.

Approximately 10–30% of pauci-immune glomerulonephritis cases are negative for ANCA [20]. Investigators have found that distinct subsets of patients with infection or malignancy can have ANCA-negative pauci-immune glomerulonephritis of secondary etiology [21]. The mechanism of ANCA-negative pauci-immune glomerulonephritis is unclear but may be due to overactivation of the alternative complement pathway [22]. Our patient's low C3 level, which appears to be a common finding in the previously reported cases listed in Table 2, suggests that a similar pathogenesis may drive kidney injury in pauci-immune glomerulonephritis associated with Bartonella endocarditis.

Current guidelines recommend doxycycline and rifampin as the preferred regimen for treating Bartonella endocarditis [23]. Our patient's initial treatment included a combination of doxycycline and rifampin, as well as vancomycin and ceftriaxone to empirically cover other possible infections causing endocarditis that could not be ruled out by the clinical team. The majority of Bartonella endocarditis patients undergo surgical removal of the infected valve; however, our patient was not a surgical candidate due to the extent of his acute kidney injury, severe malnutrition status, history of embolic strokes and hemorrhages, and severe anemia, further complicating the management of his pauci-immune glomerulonephritis. Typically, a course of pulse steroids with or without cyclophosphamide can be considered for diffuse crescentic and necrotizing infection-related glomerulonephritis when no active infection is present [24]. However, our patient's ongoing endocarditis presented a major treatment dilemma since we needed to consider whether adding immunosuppression in the form of steroids would provide a significant benefit for treating his very aggressive glomerulonephritis that would outweigh potentially worsening his Bartonella endocarditis. Ultimately, the severe activity of his glomerulonephritis favored the incorporation of steroids in the hopes of treating his kidney failure.

Our case illustrates the complex interplay between infective endocarditis and secondary kidney pathology, reinforcing the need for comprehensive diagnostic workup and a nuanced approach to management. The studies referenced provide valuable context to support the clinical decisions made in this case, while also highlighting the ongoing challenges in diagnosing and treating conditions involving multiorgan systems affected by infectious agents with concurrent inflammatory diseases.

## Figures and Tables

**Table 1:** Laboratory findings during initial and second hospitalizations.

Lab test	Result	Reference range
*Initial hospitalization*		
Blood cultures (4 sets)	Negative for growth	
Antinuclear antibodies	None detected	None detected
Anti-double-stranded DNA antibodies	21 IntUnits	24 IntUnits
Antiglomerular basement membrane antibodies	0 AU/mL	0–19 AU/mL
ANCA titer	<1 : 20	<1 : 20
Myeloperoxidase levels	Negative	
Proteinase 3 levels	Negative	
C3 level	57 mg/dL	90–180 mg/dL
C4 level	9 mg/dL	10–40 mg/dL
International normalized ratio (INR)	1.3	0.9–1.2
Prothrombin time test	29.5 seconds	22.7–34.8 seconds
Total bilirubin	0.5–0.7 mg/dL	0.2–1.2 mg/dL
Hemoglobin	5.7 g/dL	13.5–17.5 g/dL
Platelets	101 K/*µ*L	150–450 K/*µ*L
Schistocytes on peripheral smear	Present	
Lactate dehydrogenase	Not assessed	
Haptoglobin	Not assessed	
*Bartonella henselae* IgM antibody titer	1 : 128	<1 : 16
Brucella antibody titers	Negative	
Q fever antibody titers	Negative	

*Second hospitalization*		
Blood cultures (2 sets)	Negative for growth	
Bartonella henselae IgM antibody titer	1 : 32 and 1 : 16 on two subsequent tests	<1 : 16
Brucella antibody	Negative	
Total bilirubin	0.6 mg/dL	0.2–1.2 mg/dL
Hemoglobin	6.3 g/dL	13.5–17.5 g/dL
Platelets	248 K/*µ*L	150–450 K/*µ*L
International normalized ratio (INR)	1.3	0.9–1.2
Prothrombin time test	32.0 seconds	22.7–34.8 seconds

**Table 2:** Cases of Bartonella endocarditis associated with pauci-immune glomerulonephritis.

Authors	Patient age/sex	Bacterial species	Serologic studies	Biopsy findings	Treatment course	Outcomes
This case	56-year-old male	*Bartonella henselae*	ANCA negative and low C3/C4	Diffuse crescentic and necrotizing pauci-immune GN	Renal failure requiring HD. Antibiotics and steroid taper	Deceased
Shahzad et al. [16]	33-year-old male	*Bartonella henselae*	Anti-PR-3, RF, and low C4	Sclerosing pauci-immune GN with residual necrotizing lesions	Antibiotics, aortic valve replacement, and mitral valve repair	Stable 12 months after discharge
Beydon et al. [17]	78-year-old male	*Bartonella henselae*	Anti-PR3, cryoglobulinaemia, low C3, and RF	Pauci-immune crescentic GN	Antibiotics, glucocorticosteroids, cyclophosphamide, and plasmatic exchanges	Lost to follow up
Beydon et al. [17]	54-year-old male	*Bartonella henselae*	Anti-PR3, cryoglobulinaemia, low C4, and RF	Pauci-immune crescentic GN	Antibiotics, glucocorticosteroids, cyclophosphamide, and plasmatic exchanges	Good
Beydon et al. [17]	55-year-old male	*Bartonella henselae*	Anti-PR3, low C3, and RF	Pauci-immune crescentic GN	Antibiotics and glucocorticosteroids	Good
Cervi et al. [18]	59-year-old male	*Bartonella henselae*	Anti-PR3, negative p-ANCA, and low C3	Greater than 50% of glomeruli with cellular and fibrocellular crescents and some segmental sclerosis	Antibiotics and steroids	Deceased
Raybould et al. [5]	55-year-old male	*Bartonella henselae and B. quintana*	c-ANCA, negative p-ANCA, RF, low C3, and normal C4	Focal proliferative pauci-immune glomerulonephritis with rare crescents	Antibiotics and steroids	Stable at 3 months after discharge
Shah et al. [19]	36-year-old male	*Bartonella henselae*	Anti-PR3 and RF	Pauci-immune necrotizing GN	Multiple different regimens including antibiotics, steroids, and immunosuppressants. Mechanical aortic valve placement	Stable after initial worsening of condition
Vikram et al. [11]	43-year-old male	*Bartonella henselae*	PR3-ANCA	Pauci-immune pattern	Multiple different regimens including antibiotics, steroids, and immunosuppressants. Mechanical aortic and mitral valve placement	Alive 18 months after surgery

## Data Availability

The data used to support the findings of this study are available upon request.
